# Autism and ADHD social media short-form video content: A systematic review protocol

**DOI:** 10.1371/journal.pone.0357837

**Published:** 2026-09-08

**Authors:** Vasileia Karasavva, Amori Yee Mikami

**Affiliations:** University of British Columbia, Vancouver, British Columbia, Canada; KI: Karolinska Institutet, SWEDEN

## Abstract

**Rationale:**

Social media platforms have made information about mental health more accessible than ever, with over 60% of young adults using social media as a primary information source. Content about neurodiversity issues, including attention-deficit/hyperactivity disorder (ADHD) and autism spectrum disorder (ASD), is highly popular online. Researchers and mental health professionals have expressed concerns over the lack of vetting and potential spread of misinformation on social media. To date, there has been no synthesis of the literature examining the scientific accuracy of short-form video content about ADHD and ASD, and the downstream impacts of this content.

**Objectives:**

1) Describe the popularity and characteristics of ADHD and ASD short-form video content, 2) Evaluate the extent to which it follows clinical guidelines, 3) Explore its impacts on stigma, treatment- and diagnosis-seeking, and symptom perceptions.

**Methods:**

This protocol is designed in accordance with the 2020 Preferred Reporting Items for Systematic Review and Meta-Analysis Protocols (PRISMA-P) guidelines. PsycINFO, PubMed, and SCOPUS will be used for the literature search. Qualitative, quantitative, and mixed-methods peer-reviewed research published in English by June 1, 2026, will be considered. Once duplicate studies are removed, pairs of independent reviewers will determine eligibility first through the review of titles and abstracts and then full text review. Descriptive and thematic analysis methods will be used to analyze extracted data.

**Conclusion:**

Findings from this work can provide a better understanding of the social media content about ADHD and ASD and the ways it may impact viewers.

**Pre-registration:**

A protocol of this systematic literature review will be registered with Open Science Framework Registries.

## 1. Introduction

Social media has rapidly evolved from a space for connecting with friends to a central information hub where young adults learn about themselves. This includes information about mental health, which is more accessible than ever, with over 60% of young adults using social media as a primary information source [1]. Short-form video content about attention-deficit/hyperactivity disorder (ADHD) or autism spectrum disorder (ASD) symptoms is particularly popular, often ranking in the top 10 most used TikTok hashtags [2]. Some people use social media information to diagnose themselves with ADHD and/or ASD, adopting identities such as “AuDHD-er”, “ADHD-er”, and “neurodivergent” [3]. At the same time, growing concerns have been raised that the ADHD and ASD information on social media does not align with mental health professionals’ conceptualizations of these conditions. The current systematic review aims to examine the state of the knowledge about ADHD and ASD social media short-form video content, and the implications of this content. Short-form videos were initially introduced through the social media platform Vine. Although Vine no longer exists, short-form video content is now more popular than ever and almost ubiquitous on social media (e.g., TikTok, Instagram/Facebook Reels, YouTube Shorts). These videos are highly engaging and typically no longer than a couple minutes. Alongside short-form video content, scroll-based navigation and algorithmic calibration on social media platforms also became popular [4]. This means that users no longer have to actively look up content they think they will like or select from several recommendations [4]. Instead, platforms immediately serve content that a user is algorithmically predicted to like and deliver more of this content as a user scrolls, creating a continuous and seamless stream of highly engaging videos [4].

The introduction of algorithmically curated short-form videos has fundamentally changed how users engage with social media. To compete in the attention economy, platforms now prioritize visually immersive, story-driven content that fosters parasocial relationships with creators who users probably do not know in real life [5,6]. At the same time, content creators are incentivized to produce content that will yield the highest engagement metrics. These factors have led to rapid increases in both content creation and consumption, promoting public self-expression and the formation of online communities. As a result, users increasingly consume algorithmically recommended content that aligns with their existing beliefs [5]. Thus, through selective exposure, users may be placed in algorithmic silos where they perceive their views to be more widely shared than they are, thereby strengthening ideological divisions [5,7]. As well, the content consumed tends to be highly engaging, but may or may not be consistent with scientific evidence.

Young adults are among the heaviest users of social media and, as digital natives, are highly accustomed to navigating these platforms. They are also at a developmental stage where they are often making independent decisions about their mental health for the first time. Increasingly, younger generations use platforms such as TikTok as a search engine rather than relying on traditional search engines or healthcare providers [8], making them more likely to encounter mental health information that does not align with scientific evidence. In contrast, older adults may be less reliant on social media because they have established relationships with healthcare professionals (e.g., family doctors, psychiatrists, psychologists), greater financial resources, and less familiarity with navigating social media. That said, the proliferation of social media information that does not align with clinical standards is an issue that affects people across generations. For example, social media content about perimenopause, which is presumably targeted toward middle-aged adults, is also highly popular and has raised concerns about its scientific accuracy [9,10]. Thus, generational differences in online health information seeking may be less because of differences in the ability to evaluate social media health information and more due to differences in social media habits and access to traditional forms of healthcare.

Gender is also an important factor to consider in online health information seeking. Women are more likely than men to seek health information online and often serve as healthcare decision-makers not only for themselves but also for family members, including children and aging parents [11,12]. However, although women may be more likely to seek health information online, there is limited evidence of gender differences in how individuals evaluate this information [13,14]. Specifically, both men and women appear to prefer online sources for health information and do not differ substantially in their perceptions of the accuracy of the information they encounter [13,14].

Social media content about ADHD or ASD can help users feel empowered to look inward and understand their own experiences better, especially as it often centers lived experiences [15]. Such content may be especially appealing to members of historically marginalized communities who often face significant barriers in accessing mental healthcare, including financial constraints, systemic racism, and limited availability of mental health professionals who are culturally competent or share a similar background [16]. Therefore, in many ways, social media has democratized information that is otherwise hard to access or understand.

At the same time, concerns have been raised over the lack of vetting, quality control, and moderation of the mostly anecdotal user-generated content that may inadvertently introduce misinformation about the symptoms or treatment of mental health concerns, including ADHD and ASD. As was previously discussed, most major social media platforms such as TikTok, Instagram, and Facebook operate using algorithms that present users content they are likely to enjoy or agree with, with the ultimate goal of keeping them on the platform as long as possible [4]. In practice, this means that a user could be served content about ADHD and/or ASD over and over again that may or may not align with scientific evidence. Through repeated exposure, over time, users may perceive the content to be more credible, as the sleeper effect prescribes [17]. This can be compounded by confirmation bias, whereby individuals preferentially engage with information that aligns with their existing beliefs while discounting contradictory evidence, creating an echo-chamber [18].

Therefore, social media platforms have created an environment ripe for the propagation of mental health information that is deemed incomplete or misaligned with clinical guidelines by mental health professionals and researchers. There is some evidence that watching TikTok videos about ADHD could lead young adults to pathologize normative experiences or overestimate symptom severity, relative to professionals’ conceptualizations of ADHD [19,20]. In turn, mental health professionals may increasingly encounter clients whose understandings of mental health concerns such as ADHD and ASD is shaped by social media and diverges from professional conceptualizations and treatment approaches. This can result in ruptures in the therapeutic alliance and poor treatment seeking and adherence.

To date, three reviews have surveyed the empirical literature on the presentation of neurodevelopmental disorders like ADHD and/or ASD on social media [21–23]. One focused on the quality of ADHD content on TikTok and its potential to influence people to self-diagnose with ADHD [22]. The narrow focus on ADHD TikTok content and its influence on self-diagnosis could mean that cross-posted content shared on similar platforms such as Instagram Reels or YouTube Shorts, or other important impacts of such content (e.g., stigma, help-seeking, or symptom interpretation), may have been overlooked. Two more reviews explored the prevalence and quality of content on social media about mental health concerns [21] and ASD [23], as well as differences in these characteristics across platforms. However, they did not investigate the downstream effects of such content [21,23]. Therefore, a comprehensive synthesis of the existing literature on the popularity, quality, and impact of social media short-form video content about ADHD and ASD remains needed.

Accordingly, this review aims to answer three key questions: (1) how popular are ADHD and ASD short-form videos on social media? What does such content tend to look like and who posts it? (2) how scientifically accurate do researchers and mental health professionals judge social media content about ADHD and ASD to be? and (3) how does such content impact awareness and stigma about ADHD and ASD in the general population, and how do young adults who consume it view their own symptoms and make decisions about their treatment and diagnosis seeking?

## 2. Method

The protocol of this systematic review is written in accordance with the Preferred Reporting Items for Systematic Reviews and Meta-Analysis Protocols (PRISMA-P) guidelines published in 2020 (See S1 Table) [24]. Our process includes 1) inclusion and exclusion criteria, 2) search strategy, 3) study selection, 4) data extraction, 5) quality assessment, and 6) synthesis of results. The current review does not necessitate any human involvement and requires no ethical approval.

### 2.1 Eligibility criteria

#### 2.1.1 Inclusion criteria.

Included studies must: 1) be a peer-reviewed journal article published before June 1, 2026; 2) be written in English; 3) be considered an original qualitative, quantitative, or mixed methods study; 4) address at least one of the research questions.

#### 2.1.2 Exclusion criteria.

This review will exclude grey literature, books, book chapters, unpublished studies, dissertations, abstracts, and reports. In addition to addressing practical barriers like time and resource constraints, these criteria will help filter out non-peer-reviewed work, ensuring that the included studies are of higher methodological quality. Literature, systematic, or scoping reviews, and meta-analyses, will also be excluded.

Although content on ADHD and ASD is also popular on text- and image-based platforms (e.g., Reddit, Instagram), this review will focus on short-form videos for both conceptual and methodological reasons. The introduction of short-form videos was accompanied by algorithmically curated feeds based on inferred user preferences that is likely to amplify confirmation biases by repeatedly serving content with which the user is already inclined to agree. Following the rapid rise of TikTok, other platforms prioritized short-form videos, reflecting a broader shift in how users engage with social media. From a measurement perspective, including multiple different forms of social media posts (e.g., text-based, images, short- and long-form videos) would introduce a great degree of heterogeneity and would complicate attempts to synthesize findings. Thus, papers that do not examine short-form video content on social media will be excluded.

### 2.2 Search strategy

Studies will be selected through searches of the PsycINFO, PubMed, and SCOPUS databases. Additionally, the reference lists and citations of included papers as well as published systematic and scoping reviews on issues related to social media representations of ADHD and ASD will be reviewed. We will use two strings in our search combined by the *AND* Boolean operator (Table 1). An iterative process was used to develop a comprehensive keyword list. In addition to the keywords presented in Table 1, we identified a list of keywords that would address each research question (e.g., scientific accuracy for RQ2 and treatment seeking for RQ3). However, studies that were otherwise relevant were often excluded from search results when using these terms. Therefore, to maximize search sensitivity we only included terms related to neurodiversity and social media content (Table 1). Any discrepancies will be resolved through discussion and consensus to ensure consistency in study selection.

**Table 1:** Search terms.

String I	String II
“attention-deficit/hyperactivity disorder” OR “ADHD” or “Autism Spectrum Disorder” OR “ASD” or “Autism*” or “Autistic” or “Neurodiverse*” or “Neurodivergence”	“short-form videos” OR “Social Media” OR “TikTok” OR “Instagram” OR “YouTube” OR “Facebook”

#### 2.2.1 Study selection.

All identified articles will be imported into Covidence, a tool used for screening and data extraction in literature reviews, to remove duplicates and streamline the screening process. Prior to the title and abstract review, the inclusion criteria will undergo pilot testing to ensure clarity and consistency in how reviewers interpret them. To do so, reviewers will screen the first 10 articles in Covidence to see if they meet the inclusion criteria. This process will be repeated until all reviewers achieve 80% agreement. All reviewers will meet to resolve any disagreements or discuss potential improvements to the study selection process. The abstract review guidelines can be found in the Appendix (See S2 Table).

Our research team consists of graduate students working toward their PhD in clinical psychology and professors who are registered clinical psychologists with domain expertise in ADHD and/or ASD. Each article will be screened by two independent reviewers during both the title/abstract and full-text review phases. At least one reviewer in each pair will have extensive experience with ADHD and one with ASD. All reviewers will meet to resolve any disagreements at the full-text review stage.

### 2.3 Data extraction

For each selected study, we will collect the author(s), title, publication year, study design, number of participants (if applicable), number of videos examined (if applicable), study aims, and outcomes relevant to our research questions (e.g., RQ1: video metrics, RQ2: conclusions about the quality of social media ADHD and ASD content, and RQ3: impacts of this content).

Table 2 contains the information we will be extracting from all selected papers. This coding template was created by the first author following a deductive approach. All coders will code at least five articles together to ensure consistency in the application of the coding template. Disagreements will be resolved by consensus.

**Table 2:** Data extraction.

Author (Year)	Title	Study Design	Study Characteristics	RQ1	RQ2	RQ3
		• Quantitative • Qualitative • Mixed methods	• Sample size • Sample characteristics • Aims	• Video characteristics (e.g., platform posted, discussed ADHD/ASD/AuDHD, focused on treatment/assessment/symptom presentation?) • Video metrics (e.g., views, likes, shares) • Content creator characteristics (e.g., credentials)	• Credentials of raters who evaluated the content • How did raters evaluate content? • Conclusion on the quality of the content	• Impact on the individual (e.g., prognosis, perception of own symptoms, attitudes toward the healthcare system) • Societal impact (e.g., stigma, awareness, lay understanding of ADHD/ASD) • Impact on therapeutic relationship (e.g., treatment seeking and adherence)

### 2.4 Quality assessment

Two raters will independently assess the quality of included studies using the checklist in Table 3 [25]. The quality of employed methodology, results, and conclusions will be rated using a Likert scale ranging from 0 (*not addressed*) to 2 (*adequately addressed*). The two raters will rate the first five papers together to ensure consistency in the understanding and application of the quality assessment tool. Scores for qualitative studies can range from 0 to 20, while scores for quantitative studies can range from 0 to 22. Mixed-methods studies will receive separate scores for their qualitative and quantitative components, yielding a total score ranging from 0 to 42. All disagreements in scoring will be resolved collaboratively through consensus.

**Table 3:** Quality assessment checklist.

Qualitative Quality Assessment	Quantitative Quality Assessment
1. Question/objective sufficiently described	1. Question/objective sufficiently described
2. Study design evident and appropriate	2. Study design evident and appropriate
3. Context of the study clear	3. Method of subject selection described and appropriate
4. Connection to a theoretical framework/wider body of knowledge	4. Subject characteristics sufficiently described
5. Sampling strategy described, relevant, and justified	5. Outcome measure(s) well defined and robust to measurement
6. Data collection methods clearly described and systematic	6. Sample size appropriate
7. Data analysis clearly described and systematic	7. Analytic methods described/justified and appropriate
8. Use of verification procedure(s) to establish credibility	8. Some estimate of variance is reported for the main results
9. Conclusions supported by the results	9. Controlled for confounding
10. Reflexivity of the account	10. Results reported in sufficient detail
	11. Conclusions supported by the results

### 2.5 Synthesis of results

Data analysis will consist of basic descriptive analysis. Results will be organized according to the three research questions of this review and will be presented in tabular and/or diagrammatic format accompanied by a narrative summary, conducted using thematic analysis, which is typically used to highlight patterns in information [26].

## 3. Validity and reliability

All studies will be screened using predefined inclusion criteria and search terms and we will consult with a librarian to further strengthen the credibility of the search strategy. Reliability will be enhanced through independent review at the title/abstract and full-text review steps by two coders. Risk of bias will be minimized using a predetermined quality assessment checklist. Additionally, any coder who is an author on an included study will recuse themselves from its quality assessment.

## 4. Conclusion

To the best of our knowledge, this is the first systematic literature review synthesizing the empirical literature on the popularity, quality, and impacts of ADHD and ASD social media short-form video content. Findings from this work can provide valuable insights for mental health professionals about the content young adults may be exposed to online and could inform intervention strategies that better support young adults with ADHD and ASD.

## 5. Status and timeline of the study

The review is ongoing. We expect to conduct the search on June 1, 2026, and report our results within 6 months of the search.

## Supporting information

S1 Table This is the PRISMA-P checklist. (DOCX)

S2 Table This is the abstract screening guideline. (DOCX)
