# Supplementary material for: Autism and ADHD social media short-form video content: A systematic review protocol
Source: PLoS One. 2026 Sep 8;21(9):e0357837. doi: 10.1371/journal.pone.0357837 (PMC13552790; doi:10.1371/journal.pone.0357837)
Supplement: Supplementary file 2 [file pone.0357837.s002.docx]

**Appendix. Abstract Screening Guideline**

**Title**

1. Is the **title** written in English?
   a. Yes: Continue screening
   b. No: Stop screening

**Abstract Screening**

2. Is the **abstract** written in English?
   a. Yes: Continue screening
   b. No: Stop screening
3. Does the **abstract** indicate that the manuscript presents original empirical data?
   a. Yes or Unsure/Unclear: Continue screening
   b. No: Stop screening (For example, chapter, op-e, law case review, study case, theoretical paper)
4. Does the **abstract** indicate that the manuscript is a journal article?
   a. Yes or Unsure/Unclear: Continue screening
   b. No: Stop screening (For example, is it a book, book chapter, conference/proceeding paper, literature, systematic, or scoping review, meta-analysis, technical report, preprint, dissertation/thesis or a policy paper)
5. Does the **abstract** indicate that at least one of the research questions is addressed?
   a. Yes or Unsure/Unclear: Continue screening
      i. The manuscript should address at least one of the following:
         1. Description of ADHD/ASD content on social media (e.g., popularity, context, creators)
         2. Evaluation of ADHD/ASD content on social media.
         3. Examination of impact of ADHD/ASD content on the viewer, the perception of ADHD/ASD in the general population, the therapeutic relationship.
   b. No: Stop screening
6. Does the **abstract** indicate that the study uses a qualitative, quantitative, or mixed-methods design?
   a. Yes or Unsure/Unclear: Continue screening
   b. No: Stop screening

**Decision**: **Should this article be included?**

1. **Yes**, all 6 screening questions answered “Yes” or “Unsure/Unclear.”
2. **No**, at least one of the answers is a “No.”
