# Supplementary material for: Autism and ADHD social media short-form video content: A systematic review protocol
Source: PLoS One. 2026 Sep 8;21(9):e0357837. doi: 10.1371/journal.pone.0357837 (PMC13552790; doi:10.1371/journal.pone.0357837)
Supplement: Supplementary file 3 [file pone.0357837.s003.docx]

Dear Dr. Nivins,

Thank you for your review of our manuscript “*Autism and ADHD Social Media Short-form Video Content: A Systematic Review Protocol”*. We appreciate the constructive and helpful feedback from you and from the reviewer. We have made revisions and additions to the manuscript, accordingly, as detailed in the following pages.

We have addressed all comments and think that the suggestions have made our paper stronger. We hope that these revisions meet your satisfaction. However, we are certainly willing to revise the manuscript should you determine that additional revisions are necessary. Thank you for considering our revised manuscript for publication and we look forward to hearing from you.

Sincerely,

Authors

**Additional Editor Comments**

**Very nice article. Some minor suggestions as Editor with this expertise.**

*Response.* Thank you for your positive feedback and for your helpful suggestions for improvement.

Introduction:

**R1. 1. What I feel missing is that ‘How social media evolved over the years, how this evolution got converted into or actively taken part in daily life of young adults’, then these would add the background much stronger.**

*Response*. This is a great point. We agree that fleshing out the way that social media has evolved over time and what that looks like in day-to-day life is important. We have revised the Introduction accordingly:

*“Short-form videos were initially introduced through the social media platform Vine. Although Vine no longer exists, short-form video content is now more popular than ever and almost ubiquitous on social media (e.g., TikTok, Instagram/Facebook Reels, YouTube Shorts). These videos are highly engaging and typically no longer than a couple minutes. Alongside short-form video content, scroll-based navigation and algorithmic calibration on social media platforms also became popular [4]. This means that users no longer have to actively look up content they think they will like or select from several recommendations [4]. Instead, platforms immediately serve content that a user is algorithmically predicted to like and deliver more of this content as a user scrolls, creating a continuous and seamless stream of highly engaging videos [4].*

*The introduction of algorithmically curated short-form videos has fundamentally changed how users engage with social media. To compete in the attention economy, platforms now prioritize visually immersive, story-driven content that fosters parasocial relationships with creators who users probably do not know in real life [5, 6]. At the same time, content creators are incentivized to produce content that will yield the highest engagement metrics. These factors have led to rapid increases in both content creation and consumption, promoting public self-expression and the formation of online communities. As a result, users increasingly consume algorithmically recommended content that aligns with their existing beliefs [5]. Thus, through selective exposure, users may be placed in algorithmic silos where they perceive their views to be more widely shared than they are, thereby strengthening ideological divisions [5, 7]. As well, the content consumed tends to be highly engaging, but may or may not be consistent with scientific evidence.”*

**R1.2. Who are the consumers, given that age related vulnerability along with sex-specific you need to be more specific regarding the window of age.**

*Response*. Thank you for your suggestion. We agree that demographics, such as age and gender identity, are important factors to consider in regard to users’ social media habits and vulnerability to misinformation. We have added a section in our Introduction to expand on this topic:

*“Young adults are among the heaviest users of social media and, as digital natives, are highly accustomed to navigating these platforms. They are also at a developmental stage where they are often making independent decisions about their mental health for the first time. Increasingly, younger generations use platforms such as TikTok as a search engine rather than relying on traditional search engines or healthcare providers [8], making them more likely to encounter mental health information that does not align with scientific evidence. In contrast, older adults may be less reliant on social media because they have established relationships with healthcare professionals (e.g., family doctors, psychiatrists, psychologists), greater financial resources, and less familiarity with navigating social media. That said, the proliferation of social media information that does not align with clinical standards is an issue that affects people across generations. For example, social media content about perimenopause, which is presumably targeted toward middle-aged adults, is also highly popular and has raised concerns about its scientific accuracy [9, 10]. Thus, generational differences in online health information seeking may be less because of differences in the ability to evaluate social media health information and more due to differences in social media habits and access to traditional forms of healthcare.*

*Gender is also an important factor to consider in online health information seeking. Women are more likely than men to seek health information online and often serve as healthcare decision-makers not only for themselves but also for family members, including children and aging parents [11, 12]. However, although women may be more likely to seek health information online, there is limited evidence of gender differences in how individuals evaluate this information [13, 14]. Specifically, both men and women appear to prefer online sources for health information and do not differ substantially in their perceptions of the accuracy of the information they encounter [13, 14].”*

**R1.3. Add details of how different generations consumes this information from short reels \**

**and videos; and what makes specific generations get too much reliant and believe this info are true. Does it based on ‘whether these children are too early exposed to that and cannot identify what is right and wrong’.**

*Response*. Thank you for this thoughtful suggestion and for raising this interesting point. We have expanded the Introduction to acknowledge generational differences in social media use while emphasizing that susceptibility to misinformation is shaped by both platform use and access to alternative sources of information, rather than age alone. Please also see our response to R1.2, above.

*“Young adults are among the heaviest users of social media and, as digital natives, are highly accustomed to navigating these platforms. They are also at a developmental stage where they are often making independent decisions about their mental health for the first time. Increasingly, younger generations use platforms such as TikTok as a search engine rather than relying on traditional search engines or healthcare providers [8], making them more likely to encounter mental health information that does not align with scientific evidence. In contrast, older adults may be less reliant on social media because they have established relationships with healthcare professionals (e.g., family doctors, psychiatrists, psychologists), greater financial resources, and less familiarity with navigating social media. That said, the proliferation of social media information that does not align with clinical standards is an issue that affects people across generations. For example, social media content about perimenopause, which is presumably targeted toward middle-aged adults, is also highly popular and has raised concerns about its scientific accuracy [9, 10]. Thus, generational differences in online health information seeking may be less because of differences in the ability to evaluate social media health information and more due to differences in social media habits and access to traditional forms of healthcare.”*

**Reviewer Comments:**

1. Does the manuscript provide a valid rationale for the proposed study, with clearly identified and justified research questions? The research question outlined is expected to address a valid academic problem or topic and contribute to the base of knowledge in the field.

Reviewer #1: Yes

2. Is the protocol technically sound and planned in a manner that will lead to a meaningful outcome and allow testing the stated hypotheses? The manuscript should describe the methods in sufficient detail to prevent undisclosed flexibility in the experimental procedure or analysis pipeline, including sufficient outcome-neutral conditions (e.g. necessary controls, absence of floor or ceiling effects) to test the proposed hypotheses and a statistical power analysis where applicable. As there may be aspects of the methodology and analysis which can only be refined once the work is undertaken, authors should outline potential assumptions and explicitly describe what aspects of the proposed analyses, if any, are exploratory.

Reviewer #1: Yes

3. Is the methodology feasible and described in sufficient detail to allow the work to be replicable? Descriptions of methods and materials in the protocol should be reported in sufficient detail for another researcher to reproduce all experiments and analyses. The protocol should describe the appropriate controls, sample size calculations, and replication needed to ensure that the data are robust and reproducible.

Reviewer #1: Yes

4. Have the authors described where all data underlying the findings will be made available when the study is complete? The [PLOS Data policy](https://track.editorialmanager.com/CL0/https:%2F%2Fjournals.plos.org%2Fplosone%2Fs%2Fmaterials-and-software-sharing/1/010f019fa65a2e3b-31af6e5f-f8d2-4386-82d4-0620931f16d0-000000/NILvCH6y7LM8oiIfh3OXzxRDpaTGaWNaTQviwraBndg=258) requires authors to make all data underlying the findings described in their manuscript fully available without restriction, with rare exception, at the time of publication. The data should be provided as part of the manuscript or its supporting information, or deposited to a public repository. For example, in addition to summary statistics, the data points behind means, medians and variance measures should be available. If there are restrictions on publicly sharing data—e.g. participant privacy or use of data from a third party—those must be specified.

Reviewer #1: Yes

5. Is the manuscript presented in an intelligible fashion and written in standard English? PLOS ONE does not copyedit accepted manuscripts, so the language in submitted articles must be clear, correct, and unambiguous. Any typographical or grammatical errors should be corrected at revision, so please note any specific errors here.

Reviewer #1: Yes

6. Review Comments to the Author

**R2.1.  Thank you for the opportunity to review this manuscript. The proposed protocol addresses a timely and important topic by outlining a systematic review of short-form social media video content related to attention-deficit/hyperactivity disorder (ADHD) and autism spectrum disorder (ASD). The objectives are clearly defined, the protocol is well organized, and the proposed review has the potential to make a meaningful contribution to the literature. Nevertheless, several methodological inconsistencies and reporting issues should be addressed to enhance the transparency, reproducibility, and overall methodological rigor of the protocol.**

*Response.* Thank you for your comments, which have helped to improve our manuscript.

**R2.2.** **Inconsistent list of databases between the Abstract and the Methods section
The Abstract states: "PubMed, Scopus, Web of Science, and ProQuest will be used for the literature search" (p. 2, lines 29-30; the same wording also appears in the Editorial Manager submission summary). However, Section 2.2 (Search Strategy) states: "Studies will be selected through search of the PsycINFO, PubMed, and SCOPUS databases" (p. 7, lines 142-143). These are not the same set of databases-Web of Science and ProQuest are omitted, while PsycINFO is added. Because database selection directly affects the completeness and reproducibility of the review, this discrepancy should be resolved so that a single, accurate list of databases is reported consistently throughout the manuscript (Abstract, Methods, and any PRISMA-P checklist or protocol registration).**

*Response.* We greatly appreciate your attention to detail. We have edited our list of databases throughout the manuscript.

Our abstract now reads: “*PsycINFO, PubMed, and SCOPUS will be used for the literature search*.”

And 2.2. now reads: “*Studies will be selected through searches of the PsycINFO, PubMed, and SCOPUS databases.*”

**R2.3. Arithmetic inconsistency in the quality assessment scoring range
Section 2.4 states that qualitative studies can receive scores ranging from 0-20, quantitative studies from 0-22, and mixed-methods studies from 0-42 (p. 10, lines 179-182). However, Table 3 (p. 10) contains only nine qualitative assessment items and ten quantitative assessment items. Using the stated 0-2 scoring scale, the maximum possible scores would be 18 and 20, respectively, yielding a combined maximum score of 38 rather than 42.
The authors should either add the missing checklist item(s) to Table 3 (if omitted inadvertently) or revise the reported scoring ranges so that they accurately reflect the checklist presented. This is important because these score ranges are likely to be used when interpreting and reporting study quality in the completed review.**

*Response.* Thank you for pointing this out. We realize that we had omitted an item on each scale. This has now been rectified. Here are the revised scales:

| Qualitative Quality Assessment | Quantitative Quality Assessment |
| --- | --- |
| 1. Question/objective sufficiently described | 1. Question/objective sufficiently described |
| 2. Study design evident and appropriate | 2. Study design evident and appropriate |
| 3. Context of the study clear | 3. Method of subject selection described and appropriate |
| 4. Connection to a theoretical framework/wider body of knowledge | 4. Subject characteristics sufficiently described |
| 5. Sampling strategy described, relevant, and justified | 5. Outcome measure(s) well defined and robust to measurement |
| 6. Data collection methods clearly described and systematic | 6. Sample size appropriate |
| 7. Data analysis clearly described and systematic | 7. Analytic methods described/justified and appropriate |
| 8. Use of verification procedure(s) to establish credibility | 8. Some estimate of variance is reported for the main results |
| 9. Conclusions supported by the results | 9. Controlled for confounding |
| 10. Reflexivity of the account | 10. Results reported in sufficient detail |
|  | 11. Conclusions supported by the results |

**R2.4. The search strategy does not explicitly include terms related to the outcome constructs addressed in RQ2 and RQ3. Table 1 (p. 7) combines neurodevelopmental disorder terms with social media/platform terms but does not include search terms corresponding to the principal outcome constructs addressed in RQ2 and RQ3 (e.g., misinformation, scientific accuracy, guideline concordance, stigma, treatment-seeking, or symptom perception). This may represent a deliberate strategy to maximize search sensitivity while relying on manual screening to identify relevant studies. If so, the authors should briefly explain this rationale and discuss how they anticipate maintaining screening feasibility, particularly given the use of two independent reviewers and the projected six-month timeline described later in the protocol.**

*Response*. You are right about the reason why keywords related to R2 and R3 were not included. We spent a lot of time thinking about the strings for the search strategy. We piloted search terms with and without RQ2- and RQ3-related keywords and found that we increased the search sensitivity when we omitted them. We have now added this explanation in our manuscript.

“*An iterative process was used to develop a comprehensive keyword list. In addition to the keywords presented in Table 1, we identified a list of keywords that would address each research question (e.g., scientific accuracy for RQ2 and treatment seeking for RQ3). However, studies that were otherwise relevant were often excluded from search results when using these terms. Therefore, to maximize search sensitivity we only included terms related to neurodiversity and social media content (Table 1).* *Any discrepancies will be resolved through discussion and consensus to ensure consistency in study selection.”*

**R2.5. Citation supporting statements about recommendation algorithms appears incorrect
In the Introduction (p. 3), Reference 4 is cited repeatedly (lines 57-62) to support statements regarding scroll-based navigation, algorithmic feed curation, and continuous delivery of personalized content. However, Reference 4 (Chiencharoenthanakij et al.) examines associations between short-form video use and inattentive symptoms among Thai school-age children rather than recommendation algorithms or platform design. By contrast, Reference 7 (Narayanan, Understanding Social Media Recommendation Algorithms) appears to be the more appropriate citation for these statements. This appears to be a citation-management or reference-numbering error and should be corrected to ensure that the statements are supported by the appropriate source.**

*Response*. Thank you for pointing out the error with our citation manager. We have edited our citations accordingly.

**R2.6. Redundant description of supplementary searching. The manuscript describes searching the reference lists of included studies and previous reviews twice: first within the Inclusion Criteria (Section 2.1.1, p. 6, lines 121-123) and again in the Search Strategy (Section 2.2, p. 7, lines 143–145). As this represents a search procedure rather than an eligibility criterion, consider consolidating this information within the Search Strategy section.**

*Response*. We agree with this suggestion. We have edited the manuscript so that only the Search Strategy section contains the description of searching the reference lists of included studies and previous reviews.

**R2.7. Minor inconsistency in literature search dates
The Abstract states that studies published by May 30, 2026 will be included (p. 2, line 32), whereas Section 2.1.1 specifies studies published before June 2026 (p. 6, line 119), and Section 7 indicates that the search will be conducted on June 1, 2026 (p. 11, lines 208–209). Although these statements are broadly consistent, using a single, standardized cut-off date throughout the manuscript would improve clarity.**

*Response*. We have edited our language across these sections for consistency:

*“[…] published in English by June 1, 2026, will be considered.”*

*“We expect to conduct the search on June 1, 2026, and report our results within 6 months of the search.”*

*“Included studies must: 1) be a peer-reviewed journal article published before June 1, 2026;”*

**R2.8. Incomplete reference formatting. Twelve of the fifteen references (References 3, 4, and 6-15) end with the unresolved placeholder "doi." rather than an actual DOI (pp. 12-13). These appear to be artifacts of the reference-management software and should be replaced with complete DOI information where available or removed if no DOI exists.**

*Response.* Thank you for noticing this issue. We have added all missing DOIs.

**R2.9. Minor typographical and proofreading issues. Several repeated phrases and minor typographical errors suggest that the manuscript would benefit from a final proofreading. Examples include:
• p. 3, lines 46-47: "...more accessible than ever, than ever..."
• p. 5, line 95: "such us" should read "such as"
• p. 10, lines 187-188: "...which is which is typically used..."
• p. 6, line 119: "beforeJune" (missing space)**

*Response*. We are grateful that you have brought these errors to our attention. We have done another proofread to fix typing and minor language issues.

**R2.10. Consistency of citation style. Most references are cited using the journal's numerical citation style. However, the Kmet quality assessment tool is introduced as "Kmet et al. (2004)" (p. 10, line 175) without the accompanying numerical citation, despite corresponding to Reference 14. For consistency, consider using the journal's citation style uniformly throughout the manuscript.**

*Response*. We have edited all our in-text citations to be consistent with the journal’s citation style.
