# Supplementary material for: Autism and ADHD social media short-form video content: A systematic review protocol
Source: PLoS One. 2026 Sep 8;21(9):e0357837. doi: 10.1371/journal.pone.0357837 (PMC13552790; doi:10.1371/journal.pone.0357837)
Supplement: Peer review history

# Peer review: Decision Letter 0

**DOI:** 10.1371/journal.pone.0357837.r001

**Authors:** Samson Nivins

27 Jul 2026

Thank you for submitting your manuscript to PLOS ONE. After careful consideration, we feel that it has merit but does not fully meet PLOS ONE’s publication criteria as it currently stands. Therefore, we invite you to submit a revised version of the manuscript that addresses the points raised during the review process.

Please submit your revised manuscript by Sep 10 2026 11:59PM. If you will need more time than this to complete your revisions, please reply to this message or contact the journal office at plosone@plos.org. When you're ready to submit your revision, log on to https://www.editorialmanager.com/pone/ and select the 'Submissions Needing Revision' folder to locate your manuscript file.

- A letter that responds to each point raised by the academic editor and reviewer(s). You should upload this letter as a separate file labeled 'Response to Reviewers'.
- A marked-up copy of your manuscript that highlights changes made to the original version. You should upload this as a separate file labeled 'Revised Manuscript with Track Changes'.
- An unmarked version of your revised paper without tracked changes. You should upload this as a separate file labeled 'Manuscript'.

If you would like to make changes to your financial disclosure, please include your updated statement in your cover letter. Guidelines for resubmitting your figure files are available below the reviewer comments at the end of this letter.

If applicable, we recommend that you deposit your laboratory protocols in protocols.io to enhance the reproducibility of your results. Protocols.io assigns your protocol its own identifier (DOI) so that it can be cited independently in the future. For instructions see: https://journals.plos.org/plosone/s/submission-guidelines#loc-laboratory-protocols. Additionally, PLOS ONE offers an option for publishing peer-reviewed Lab Protocol articles, which describe protocols hosted on protocols.io. Read more information on sharing protocols at https://plos.org/protocols?utm_medium=editorial-email&utm_source=authorletters&utm_campaign=protocols.

As the corresponding author, your ORCID iD is verified in the submission system and will appear in the published article. PLOS supports the use of ORCID, and we encourage all coauthors to register for an ORCID iD and use it as well. Please encourage your coauthors to verify their ORCID iD within the submission system before final acceptance, as unverified ORCID iDs will not appear in the published article. *Only*  the individual author can complete the verification step; PLOS staff *cannot*  verify ORCID iDs on behalf of authors.

We look forward to receiving your revised manuscript.

Kind regards,

Samson Nivins, Ph.D

Academic Editor

PLOS One

**Journal Requirements:**

1. When submitting your revision, we need you to address these additional requirements.

Please ensure that your manuscript meets PLOS ONE's style requirements, including those for file naming. The PLOS ONE style templates can be found at

https://journals.plos.org/plosone/s/file?id=wjVg/PLOSOne_formatting_sample_main_body.pdf and

https://journals.plos.org/plosone/s/file?id=ba62/PLOSOne_formatting_sample_title_authors_affiliations.pdf

2. Please include captions for your Supporting Information files at the end of your manuscript, and update any in-text citations to match accordingly. Please see our Supporting Information guidelines for more information: http://journals.plos.org/plosone/s/supporting-information.

3. If the reviewer comments include a recommendation to cite specific previously published works, please review and evaluate these publications to determine whether they are relevant and should be cited. There is no requirement to cite these works unless the editor has indicated otherwise. 

4. Please review your reference list to ensure that it is complete and correct. If you have cited papers that have been retracted, please include the rationale for doing so in the manuscript text, or remove these references and replace them with relevant current references. Any changes to the reference list should be mentioned in the rebuttal letter that accompanies your revised manuscript. If you need to cite a retracted article, indicate the article’s retracted status in the References list and also include a citation and full reference for the retraction notice.

**Additional Editor Comments:**

Introduction:

What I feel missing is that ‘How social media evolved over the years, how this evolution got converted into or actively taken part in daily life of young adults’, then these would add the background much stronger.

Who are the consumers, given that age related vulnerability along with sex-specific you need to be more specific regarding the window of age

Add details of how different generations consumes this information from short reels and videos; and what makes specific generations get too much reliant and believe this info are true. Does it based on ‘whether these children are too early exposed to that and cannot identify what is right and wrong’.

[Note: HTML markup is below. Please do not edit.]

Reviewers' comments:

Reviewer's Responses to Questions

**Comments to the Author**

1. Does the manuscript provide a valid rationale for the proposed study, with clearly identified and justified research questions?

Reviewer #1: Yes

**********

2. Is the protocol technically sound and planned in a manner that will lead to a meaningful outcome and allow testing the stated hypotheses?

Reviewer #1: Yes

**********

3. Is the methodology feasible and described in sufficient detail to allow the work to be replicable?

Reviewer #1: Yes

**********

4. Have the authors described where all data underlying the findings will be made available when the study is complete?

The PLOS Data policy

Reviewer #1: Yes

**********

5. Is the manuscript presented in an intelligible fashion and written in standard English?

Reviewer #1: Yes

**********

Please use the space provided to explain your answers to the questions above and, if applicable, provide comments about issues authors must address before this protocol can be accepted for publication. You may also include additional comments for the author, including concerns about research or publication ethics.

You may also provide optional suggestions and comments to authors that they might find helpful in planning their study.

**Reviewer #1:**  Thank you for the opportunity to review this manuscript. The proposed protocol addresses a timely and important topic by outlining a systematic review of short-form social media video content related to attention-deficit/hyperactivity disorder (ADHD) and autism spectrum disorder (ASD). The objectives are clearly defined, the protocol is well organized, and the proposed review has the potential to make a meaningful contribution to the literature. Nevertheless, several methodological inconsistencies and reporting issues should be addressed to enhance the transparency, reproducibility, and overall methodological rigor of the protocol.

Inconsistent list of databases between the Abstract and the Methods section

The Abstract states: "PubMed, Scopus, Web of Science, and ProQuest will be used for the literature search" (p. 2, lines 29-30; the same wording also appears in the Editorial Manager submission summary). However, Section 2.2 (Search Strategy) states: "Studies will be selected through search of the PsycINFO, PubMed, and SCOPUS databases" (p. 7, lines 142-143). These are not the same set of databases-Web of Science and ProQuest are omitted, while PsycINFO is added. Because database selection directly affects the completeness and reproducibility of the review, this discrepancy should be resolved so that a single, accurate list of databases is reported consistently throughout the manuscript (Abstract, Methods, and any PRISMA-P checklist or protocol registration).

Arithmetic inconsistency in the quality assessment scoring range

Section 2.4 states that qualitative studies can receive scores ranging from 0-20, quantitative studies from 0-22, and mixed-methods studies from 0-42 (p. 10, lines 179-182). However, Table 3 (p. 10) contains only nine qualitative assessment items and ten quantitative assessment items. Using the stated 0-2 scoring scale, the maximum possible scores would be 18 and 20, respectively, yielding a combined maximum score of 38 rather than 42.

The authors should either add the missing checklist item(s) to Table 3 (if omitted inadvertently) or revise the reported scoring ranges so that they accurately reflect the checklist presented. This is important because these score ranges are likely to be used when interpreting and reporting study quality in the completed review.

The search strategy does not explicitly include terms related to the outcome constructs addressed in RQ2 and RQ3

Table 1 (p. 7) combines neurodevelopmental disorder terms with social media/platform terms but does not include search terms corresponding to the principal outcome constructs addressed in RQ2 and RQ3 (e.g., misinformation, scientific accuracy, guideline concordance, stigma, treatment-seeking, or symptom perception).

This may represent a deliberate strategy to maximize search sensitivity while relying on manual screening to identify relevant studies. If so, the authors should briefly explain this rationale and discuss how they anticipate maintaining screening feasibility, particularly given the use of two independent reviewers and the projected six-month timeline described later in the protocol.

Citation supporting statements about recommendation algorithms appears incorrect

In the Introduction (p. 3), Reference 4 is cited repeatedly (lines 57-62) to support statements regarding scroll-based navigation, algorithmic feed curation, and continuous delivery of personalized content. However, Reference 4 (Chiencharoenthanakij et al.) examines associations between short-form video use and inattentive symptoms among Thai school-age children rather than recommendation algorithms or platform design.

By contrast, Reference 7 (Narayanan, Understanding Social Media Recommendation Algorithms) appears to be the more appropriate citation for these statements. This appears to be a citation-management or reference-numbering error and should be corrected to ensure that the statements are supported by the appropriate source.

Redundant description of supplementary searching

The manuscript describes searching the reference lists of included studies and previous reviews twice: first within the Inclusion Criteria (Section 2.1.1, p. 6, lines 121-123) and again in the Search Strategy (Section 2.2, p. 7, lines 143–145). As this represents a search procedure rather than an eligibility criterion, consider consolidating this information within the Search Strategy section.

Minor inconsistency in literature search dates

The Abstract states that studies published by May 30, 2026 will be included (p. 2, line 32), whereas Section 2.1.1 specifies studies published before June 2026 (p. 6, line 119), and Section 7 indicates that the search will be conducted on June 1, 2026 (p. 11, lines 208–209). Although these statements are broadly consistent, using a single, standardized cut-off date throughout the manuscript would improve clarity.

Incomplete reference formatting

Twelve of the fifteen references (References 3, 4, and 6-15) end with the unresolved placeholder "doi." rather than an actual DOI (pp. 12-13). These appear to be artifacts of the reference-management software and should be replaced with complete DOI information where available or removed if no DOI exists.

Minor typographical and proofreading issues

Several repeated phrases and minor typographical errors suggest that the manuscript would benefit from a final proofreading. Examples include:

• p. 3, lines 46-47: "...more accessible than ever, than ever..."

• p. 5, line 95: "such us" should read "such as"

• p. 10, lines 187-188: "...which is which is typically used..."

• p. 6, line 119: "beforeJune" (missing space)

Consistency of citation style

Most references are cited using the journal's numerical citation style. However, the Kmet quality assessment tool is introduced as "Kmet et al. (2004)" (p. 10, line 175) without the accompanying numerical citation, despite corresponding to Reference 14. For consistency, consider using the journal's citation style uniformly throughout the manuscript.

**********

what does this mean?). If published, this will include your full peer review and any attached files.

If you choose “no”, your identity will remain anonymous but your review may still be made public.

**Do you want your identity to be public for this peer review?**  For information about this choice, including consent withdrawal, please see our Privacy Policy

Reviewer #1: No

**********

[NOTE: If reviewer comments were submitted as an attachment file, they will be attached to this email and accessible via the submission site. Please log into your account, locate the manuscript record, and check for the action link "View Attachments". If this link does not appear, there are no attachment files.]

To ensure your figures meet our technical requirements, please review our figure guidelines: https://journals.plos.org/plosone/s/figures

You may also use PLOS’s free figure tool, NAAS, to help you prepare publication quality figures: https://journals.plos.org/plosone/s/figures#loc-tools-for-figure-preparation.

NAAS will assess whether your figures meet our technical requirements by comparing each figure against our figure specifications.

# Author comment: Author response to Decision Letter 1

**DOI:** 10.1371/journal.pone.0357837.r002

10 Aug 2026

Additional Editor Comments

Very nice article. Some minor suggestions as Editor with this expertise.

Response. Thank you for your positive feedback and for your helpful suggestions for improvement.

Introduction:

R1. 1. What I feel missing is that ‘How social media evolved over the years, how this evolution got converted into or actively taken part in daily life of young adults’, then these would add the background much stronger.

Response. This is a great point. We agree that fleshing out the way that social media has evolved over time and what that looks like in day-to-day life is important. We have revised the Introduction accordingly:

“Short-form videos were initially introduced through the social media platform Vine. Although Vine no longer exists, short-form video content is now more popular than ever and almost ubiquitous on social media (e.g., TikTok, Instagram/Facebook Reels, YouTube Shorts). These videos are highly engaging and typically no longer than a couple minutes. Alongside short-form video content, scroll-based navigation and algorithmic calibration on social media platforms also became popular [4]. This means that users no longer have to actively look up content they think they will like or select from several recommendations [4]. Instead, platforms immediately serve content that a user is algorithmically predicted to like and deliver more of this content as a user scrolls, creating a continuous and seamless stream of highly engaging videos [4].

The introduction of algorithmically curated short-form videos has fundamentally changed how users engage with social media. To compete in the attention economy, platforms now prioritize visually immersive, story-driven content that fosters parasocial relationships with creators who users probably do not know in real life [5, 6]. At the same time, content creators are incentivized to produce content that will yield the highest engagement metrics. These factors have led to rapid increases in both content creation and consumption, promoting public self-expression and the formation of online communities. As a result, users increasingly consume algorithmically recommended content that aligns with their existing beliefs [5]. Thus, through selective exposure, users may be placed in algorithmic silos where they perceive their views to be more widely shared than they are, thereby strengthening ideological divisions [5, 7]. As well, the content consumed tends to be highly engaging, but may or may not be consistent with scientific evidence.”

R1.2. Who are the consumers, given that age related vulnerability along with sex-specific you need to be more specific regarding the window of age.

Response. Thank you for your suggestion. We agree that demographics, such as age and gender identity, are important factors to consider in regard to users’ social media habits and vulnerability to misinformation. We have added a section in our Introduction to expand on this topic:

“Young adults are among the heaviest users of social media and, as digital natives, are highly accustomed to navigating these platforms. They are also at a developmental stage where they are often making independent decisions about their mental health for the first time. Increasingly, younger generations use platforms such as TikTok as a search engine rather than relying on traditional search engines or healthcare providers [8], making them more likely to encounter mental health information that does not align with scientific evidence. In contrast, older adults may be less reliant on social media because they have established relationships with healthcare professionals (e.g., family doctors, psychiatrists, psychologists), greater financial resources, and less familiarity with navigating social media. That said, the proliferation of social media information that does not align with clinical standards is an issue that affects people across generations. For example, social media content about perimenopause, which is presumably targeted toward middle-aged adults, is also highly popular and has raised concerns about its scientific accuracy [9, 10]. Thus, generational differences in online health information seeking may be less because of differences in the ability to evaluate social media health information and more due to differences in social media habits and access to traditional forms of healthcare.

Gender is also an important factor to consider in online health information seeking. Women are more likely than men to seek health information online and often serve as healthcare decision-makers not only for themselves but also for family members, including children and aging parents [11, 12]. However, although women may be more likely to seek health information online, there is limited evidence of gender differences in how individuals evaluate this information [13, 14]. Specifically, both men and women appear to prefer online sources for health information and do not differ substantially in their perceptions of the accuracy of the information they encounter [13, 14].”

R1.3. Add details of how different generations consumes this information from short reels \ and videos; and what makes specific generations get too much reliant and believe this info are true. Does it based on ‘whether these children are too early exposed to that and cannot identify what is right and wrong’.

Response. Thank you for this thoughtful suggestion and for raising this interesting point. We have expanded the Introduction to acknowledge generational differences in social media use while emphasizing that susceptibility to misinformation is shaped by both platform use and access to alternative sources of information, rather than age alone. Please also see our response to R1.2, above.

“Young adults are among the heaviest users of social media and, as digital natives, are highly accustomed to navigating these platforms. They are also at a developmental stage where they are often making independent decisions about their mental health for the first time. Increasingly, younger generations use platforms such as TikTok as a search engine rather than relying on traditional search engines or healthcare providers [8], making them more likely to encounter mental health information that does not align with scientific evidence. In contrast, older adults may be less reliant on social media because they have established relationships with healthcare professionals (e.g., family doctors, psychiatrists, psychologists), greater financial resources, and less familiarity with navigating social media. That said, the proliferation of social media information that does not align with clinical standards is an issue that affects people across generations. For example, social media content about perimenopause, which is presumably targeted toward middle-aged adults, is also highly popular and has raised concerns about its scientific accuracy [9, 10]. Thus, generational differences in online health information seeking may be less because of differences in the ability to evaluate social media health information and more due to differences in social media habits and access to traditional forms of healthcare.”

Reviewer Comments:

1. Does the manuscript provide a valid rationale for the proposed study, with clearly identified and justified research questions? The research question outlined is expected to address a valid academic problem or topic and contribute to the base of knowledge in the field.

Reviewer #1: Yes

2. Is the protocol technically sound and planned in a manner that will lead to a meaningful outcome and allow testing the stated hypotheses? The manuscript should describe the methods in sufficient detail to prevent undisclosed flexibility in the experimental procedure or analysis pipeline, including sufficient outcome-neutral conditions (e.g. necessary controls, absence of floor or ceiling effects) to test the proposed hypotheses and a statistical power analysis where applicable. As there may be aspects of the methodology and analysis which can only be refined once the work is undertaken, authors should outline potential assumptions and explicitly describe what aspects of the proposed analyses, if any, are exploratory.

Reviewer #1: Yes

3. Is the methodology feasible and described in sufficient detail to allow the work to be replicable? Descriptions of methods and materials in the protocol should be reported in sufficient detail for another researcher to reproduce all experiments and analyses. The protocol should describe the appropriate controls, sample size calculations, and replication needed to ensure that the data are robust and reproducible.

Reviewer #1: Yes

4. Have the authors described where all data underlying the findings will be made available when the study is complete? The PLOS Data policy requires authors to make all data underlying the findings described in their manuscript fully available without restriction, with rare exception, at the time of publication. The data should be provided as part of the manuscript or its supporting information, or deposited to a public repository. For example, in addition to summary statistics, the data points behind means, medians and variance measures should be available. If there are restrictions on publicly sharing data—e.g. participant privacy or use of data from a third party—those must be specified.

Reviewer #1: Yes

5. Is the manuscript presented in an intelligible fashion and written in standard English? PLOS ONE does not copyedit accepted manuscripts, so the language in submitted articles must be clear, correct, and unambiguous. Any typographical or grammatical errors should be corrected at revision, so please note any specific errors here.

Reviewer #1: Yes

6. Review Comments to the Author

R2.1. Thank you for the opportunity to review this manuscript. The proposed protocol addresses a timely and important topic by outlining a systematic review of short-form social media video content related to attention-deficit/hyperactivity disorder (ADHD) and autism spectrum disorder (ASD). The objectives are clearly defined, the protocol is well organized, and the proposed review has the potential to make a meaningful contribution to the literature. Nevertheless, several methodological inconsistencies and reporting issues should be addressed to enhance the transparency, reproducibility, and overall methodological rigor of the protocol.

Response. Thank you for your comments, which have helped to improve our manuscript.

R2.2. Inconsistent list of databases between the Abstract and the Methods section

The Abstract states: "PubMed, Scopus, Web of Science, and ProQuest will be used for the literature search" (p. 2, lines 29-30; the same wording also appears in the Editorial Manager submission summary). However, Section 2.2 (Search Strategy) states: "Studies will be selected through search of the PsycINFO, PubMed, and SCOPUS databases" (p. 7, lines 142-143). These are not the same set of databases-Web of Science and ProQuest are omitted, while PsycINFO is added. Because database selection directly affects the completeness and reproducibility of the review, this discrepancy should be resolved so that a single, accurate list of databases is reported consistently throughout the manuscript (Abstract, Methods, and any PRISMA-P checklist or protocol registration).

Response. We greatly appreciate your attention to detail. We have edited our list of databases throughout the manuscript.

Our abstract now reads: “PsycINFO, PubMed, and SCOPUS will be used for the literature search.”

And 2.2. now reads: “Studies will be selected through searches of the PsycINFO, PubMed, and SCOPUS databases.”

R2.3. Arithmetic inconsistency in the quality assessment scoring range

Section 2.4 states that qualitative studies can receive scores ranging from 0-20, quantitative studies from 0-22, and mixed-methods studies from 0-42 (p. 10, lines 179-182). However, Table 3 (p. 10) contains only nine qualitative assessment items and ten quantitative assessment items. Using the stated 0-2 scoring scale, the maximum possible scores would be 18 and 20, respectively, yielding a combined maximum score of 38 rather than 42.

The authors should either add the missing checklist item(s) to Table 3 (if omitted inadvertently) or revise the reported scoring ranges so that they accurately reflect the checklist presented. This is important because these score ranges are likely to be used when interpreting and reporting study quality in the completed review.

Response. Thank you for pointing this out. We realize that we had omitted an item on each scale. This has now been rectified. Here are the revised scales:

Qualitative Quality Assessment Quantitative Quality Assessment

1. Question/objective sufficiently described 1. Question/objective sufficiently described

2. Study design evident and appropriate 2. Study design evident and appropriate

3. Context of the study clear 3. Method of subject selection described and appropriate

4. Connection to a theoretical framework/wider body of knowledge 4. Subject characteristics sufficiently described

5. Sampling strategy described, relevant, and justified 5. Outcome measure(s) well defined and robust to measurement

6. Data collection methods clearly described and systematic 6. Sample size appropriate

7. Data analysis clearly described and systematic 7. Analytic methods described/justified and appropriate

8. Use of verification procedure(s) to establish credibility 8. Some estimate of variance is reported for the main results

9. Conclusions supported by the results 9. Controlled for confounding

10. Reflexivity of the account 10. Results reported in sufficient detail

11. Conclusions supported by the results

R2.4. The search strategy does not explicitly include terms related to the outcome constructs addressed in RQ2 and RQ3. Table 1 (p. 7) combines neurodevelopmental disorder terms with social media/platform terms but does not include search terms corresponding to the principal outcome constructs addressed in RQ2 and RQ3 (e.g., misinformation, scientific accuracy, guideline concordance, stigma, treatment-seeking, or symptom perception). This may represent a deliberate strategy to maximize search sensitivity while relying on manual screening to identify relevant studies. If so, the authors should briefly explain this rationale and discuss how they anticipate maintaining screening feasibility, particularly given the use of two independent reviewers and the projected six-month timeline described later in the protocol.

Response. You are right about the reason why keywords related to R2 and R3 were not included. We spent a lot of time thinking about the strings for the search strategy. We piloted search terms with and without RQ2- and RQ3-related keywords and found that we increased the search sensitivity when we omitted them. We have now added this explanation in our manuscript.

“An iterative process was used to develop a comprehensive keyword list. In addition to the keywords presented in Table 1, we identified a list of keywords that would address each research question (e.g., scientific accuracy for RQ2 and treatment seeking for RQ3). However, studies that were otherwise relevant were often excluded from search results when using these terms. Therefore, to maximize search sensitivity we only included terms related to neurodiversity and social media content (Table 1). Any discrepancies will be resolved through discussion and consensus to ensure consistency in study selection.”

R2.5. Citation supporting statements about recommendation algorithms appears incorrect

In the Introduction (p. 3), Reference 4 is cited repeatedly (lines 57-62) to support statements regarding scroll-based navigation, algorithmic feed curation, and continuous delivery of personalized content. However, Reference 4 (Chiencharoenthanakij et al.) examines associations between short-form video use and inattentive symptoms among Thai school-age children rather than recommendation algorithms or platform design. By contrast, Reference 7 (Narayanan, Understanding Social Media Recommendation Algorithms) appears to be the more appropriate citation for these statements. This appears to be a citatio

Attachment Submitted filename: Response to Reviewers.docx

# Editor report: Decision Letter 1

**DOI:** 10.1371/journal.pone.0357837.r003

**Authors:** Samson Nivins

23 Aug 2026

Autism and ADHD Social Media Short-form Video Content: A Systematic Review Protocol

PONE-D-26-15282R1

Dear Dr. Karasavva,

We’re pleased to inform you that your manuscript has been judged scientifically suitable for publication and will be formally accepted for publication once it meets all outstanding technical requirements.

Within one week, you’ll receive an e-mail detailing the required amendments. When these have been addressed, you’ll receive a formal acceptance letter and your manuscript will be scheduled for publication.

An invoice will be generated when your article is formally accepted. Please note, if your institution has a publishing partnership with PLOS and your article meets the relevant criteria, all or part of your publication costs will be covered. Please make sure your user information is up-to-date by logging into Editorial Manager at Editorial Manager® and clicking the ‘Update My Information' link at the top of the page. For questions related to billing, please contact billing support.

If your institution or institutions have a press office, please notify them about your upcoming paper to help maximize its impact. If they’ll be preparing press materials, please inform our press team as soon as possible -- no later than 48 hours after receiving the formal acceptance. Your manuscript will remain under strict press embargo until 2 pm Eastern Time on the date of publication. For more information, please contact onepress@plos.org.

Kind regards,

Samson Nivins, Ph.D

Academic Editor

PLOS One

Additional Editor Comments (optional):

All comments are adequately addressed.

Reviewers' comments:

# Editor report: Acceptance letter

**DOI:** 10.1371/journal.pone.0357837.r004

**Authors:** Samson Nivins

PONE-D-26-15282R1

PLOS One

Dear Dr. Karasavva,

I'm pleased to inform you that your manuscript has been deemed suitable for publication in PLOS One. Congratulations! Your manuscript is now being handed over to our production team.

At this stage, our production department will prepare your paper for publication. This includes ensuring the following:

* All references, tables, and figures are properly cited

* All relevant supporting information is included in the manuscript submission,

* There are no issues that prevent the paper from being properly typeset

You will receive further instructions from the production team, including instructions on how to review your proof when it is ready. Please keep in mind that we are working through a large volume of accepted articles, so please give us a few days to review your paper and let you know the next and final steps.

Lastly, if your institution or institutions have a press office, please let them know about your upcoming paper now to help maximize its impact. If they'll be preparing press materials, please inform our press team within the next 48 hours. Your manuscript will remain under strict press embargo until 2 pm Eastern Time on the date of publication. For more information, please contact onepress@plos.org.

You will receive an invoice from PLOS for your publication fee after your manuscript has reached the completed accept phase. If you receive an email requesting payment before acceptance or for any other service, this may be a phishing scheme. Learn how to identify phishing emails and protect your accounts at https://explore.plos.org/phishing.

If we can help with anything else, please email us at customercare@plos.org.

Thank you for submitting your work to PLOS One and supporting open access.

Kind regards,

PLOS One Editorial Office Staff

on behalf of

Dr. Samson Nivins

Academic Editor

PLOS One
